# Supplementary figures and images for: Depressive symptoms, functional impairment, and health-related quality of life in idiopathic normal pressure hydrocephalus: A population-based study
Source: PLoS One. 2024 Jul 30;19(7):e0308079. doi: 10.1371/journal.pone.0308079 (PMC11288432; doi:10.1371/journal.pone.0308079)

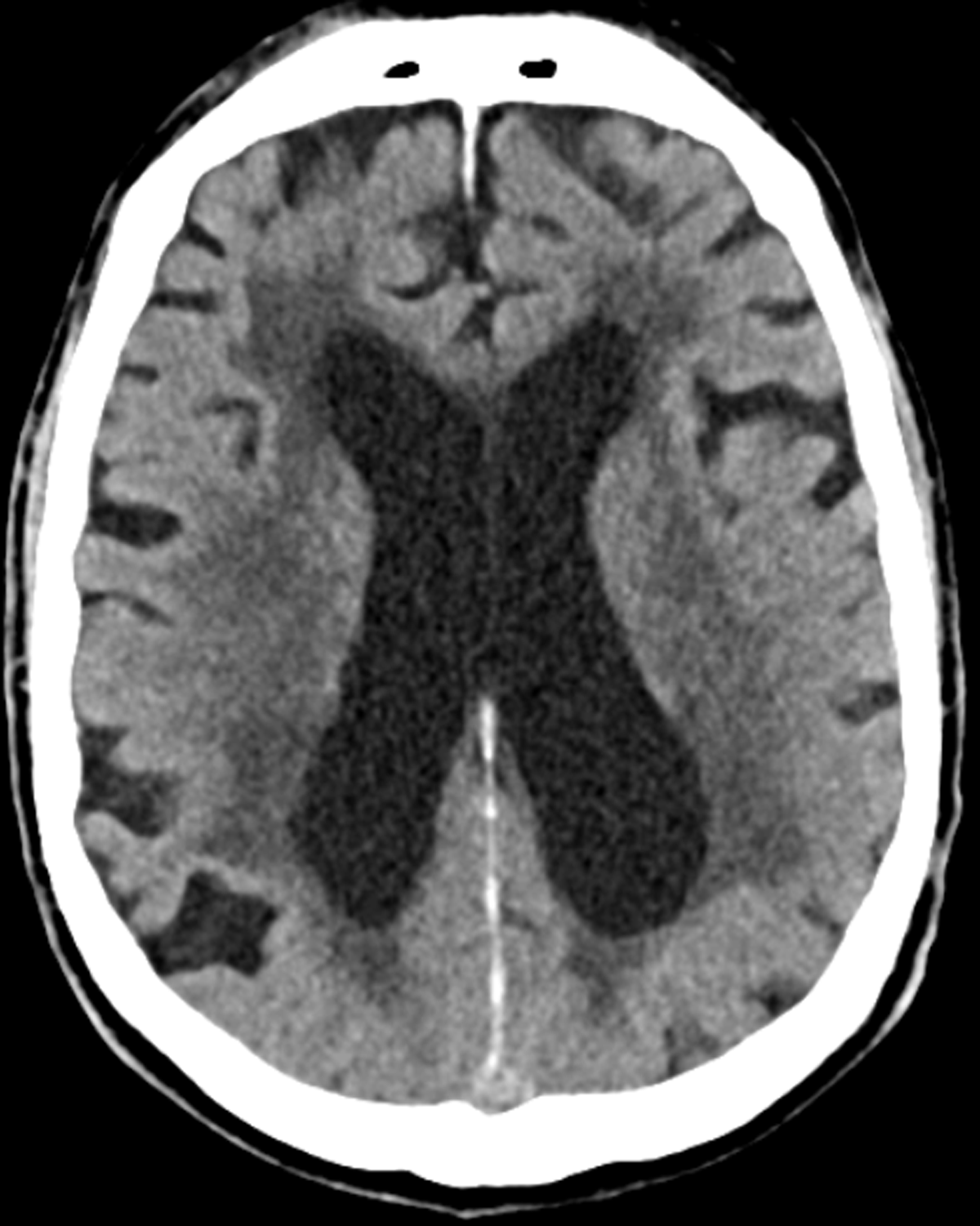

Supplement: S1 Fig — Ventriculomegali, Evans’ index > 0.30. (TIF) [file pone.0308079.s001.tif]

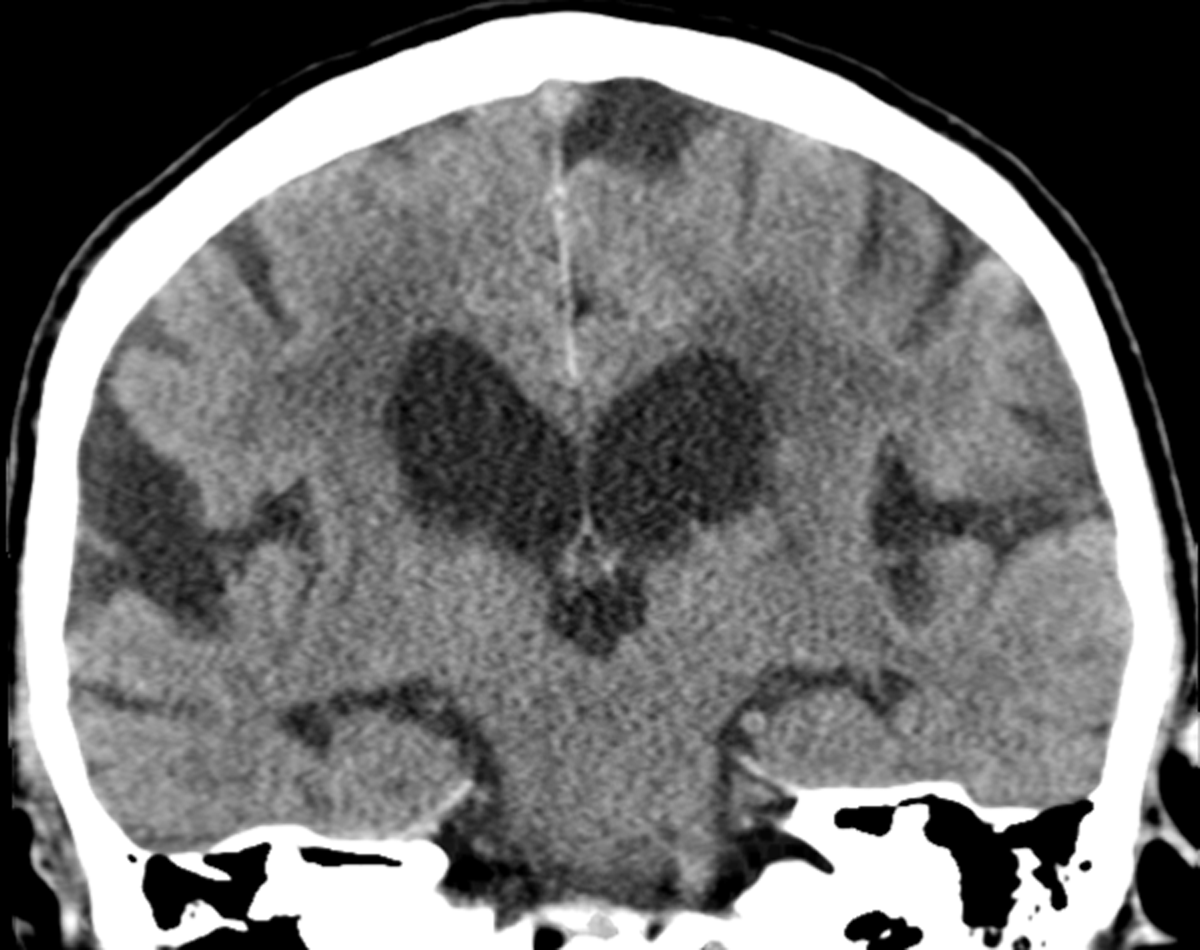

Supplement: S2 Fig — DESH/ Widening of Sylvian fissures, combined with narrowing of the sulci over the high convexity. (TIF) [file pone.0308079.s002.tif]
