# Supplementary material for: Depressive symptoms, functional impairment, and health-related quality of life in idiopathic normal pressure hydrocephalus: A population-based study
Source: PLoS One. 2024 Jul 30;19(7):e0308079. doi: 10.1371/journal.pone.0308079 (PMC11288432; doi:10.1371/journal.pone.0308079)
Supplement: Attachment — Submitted filename: Revised response to reviewers.docx [file pone.0308079.s003.docx]

**Response to reviewers**

*Dear editors and reviewers,*

*Thank you for your time and valuable comments that we believe improved the manuscript.*

*In this document we have tried to address your points. See headlines “****Author response****”,* *with the response written in italics.*

*The revised cover letter includes an amended funding statement and a further clarification regarding the issue of dual publication.*

Journal Requirements:

When submitting your revision, we need you to address these additional requirements.

1. Please ensure that your manuscript meets PLOS ONE's style requirements, including those for file naming. The PLOS ONE style templates can be found at 
https://journals.plos.org/plosone/s/file?id=wjVg/PLOSOne_formatting_sample_main_body.pdf and 
https://journals.plos.org/plosone/s/file?id=ba62/PLOSOne_formatting_sample_title_authors_affiliations.pdf

2. Note from Emily Chenette, Editor in Chief of PLOS ONE, and Iain Hrynaszkiewicz, Director of Open Research Solutions at PLOS: Did you know that depositing data in a repository is associated with up to a 25% citation advantage (https://doi.org/10.1371/journal.pone.0230416)? If you’ve not already done so, consider depositing your raw data in a repository to ensure your work is read, appreciated and cited by the largest possible audience. You’ll also earn an Accessible Data icon on your published paper if you deposit your data in any participating repository (<https://plos.org/open-science/open-data/#accessible-data>).

***Author response:*** *Thank you for reminding us about this opportunity, however public availability would compromise the participants' privacy. This is due to data restriction policies and due to what has been stated in the “informed consent” information to the participants, which has also been approved by the regional ethical board. For more information, see comments under point 5, Journal Requirements.*

3. Thank you for stating the following in the Acknowledgments Section of your manuscript: 
"We would like to thank region Jämtland-Härjedalen for funding the study, the staff at the neurological and radiological departments at Östersund’s hospital for their administrative and technical assistance. Finally, we would like to express our deepest gratitude to all the study participants for joining the study."

We note that you have provided funding information that is not currently declared in your Funding Statement. However, funding information should not appear in the Acknowledgments section or other areas of your manuscript. We will only publish funding information present in the Funding Statement section of the online submission form. 
Please remove any funding-related text from the manuscript and let us know how you would like to update your Funding Statement. Currently, your Funding Statement reads as follows: 
"KL, JA, MM received governmental funding from Region Jämtland Härjedalen. JA received funding from the donation funds of "Syskonen Perssons donations fond" , "Jämtlands cancer och omvårdnads fond" and "Stiftelsen Forskningsfonden för klinisk neurovetenskap vid NUS".

The funders had no role in study design, data collection and analysis, decision to publish, or preparation of the manuscript."

Please include your amended statements within your cover letter; we will change the online submission form on your behalf.

***Author response:*** *Thank you for this comment, we have addressed this issue with a change in the manuscript, see page 20, line 412-413. The correct funding statement is included in the revised cover letter.*

4.We noted in your submission details that a portion of your manuscript may have been presented or published elsewhere. [Yes, a previous version of this article was a part of my dissertation. It has not previously been published elsewhere. Therefore it does not constitute dual publication.

Links and identification id regarding this dissertation below:

https://www.diva-portal.org/smash/record.jsf?pid=diva2%3A1611256&dswid=-1288

URN: urn:nbn:se:umu:diva-189510
ISBN: 978-91-7855-652-6 (tryckt)
ISBN: 978-91-7855-653-3 (digital)
OAI: oai:DiVA.org:umu-189510
DiVA, id: diva2:1611256] Please clarify whether this [conference proceeding or publication] was peer-reviewed and formally published. If this work was previously peer-reviewed and published, in the cover letter please provide the reason that this work does not constitute dual publication and should be included in the current manuscript.

***Author response:*** *An early version of the manuscript was published in Johanna Andersson's doctoral thesis. Even though the thesis is a printed book (also available in digital form) the article has not been formally peer-reviewed or published in a scientific journal. Therefore, we do not consider this a dual publication.*

5. In the online submission form, you indicated that [Data are available on request, provided that approval from the Regional Ethics
Committee is given. This is because it was stated in the application to the ethical
board, as well as in the information to the study participants, that data are kept
confidential and only accessible for the researchers responsible for the study.
Requests may be sent to the corresponding author or to the Regional Ethics
Committee, Umeå. The address for such a request is:
Regionala Etikprövningsnämnden i Umeå,
Samverkanshuset, C/o Umeå Universitet, 901 87
UMEÅ SWEDEN
E-mail: epn@adm.umu.se]. 
All PLOS journals now require all data underlying the findings described in their manuscript to be freely available to other researchers, either 1. In a public repository, 2. Within the manuscript itself, or 3. Uploaded as supplementary information.
This policy applies to all data except where public deposition would breach compliance with the protocol approved by your research ethics board. If your data cannot be made publicly available for ethical or legal reasons (e.g., public availability would compromise patient privacy), please explain your reasons on resubmission and your exemption request will be escalated for approval.

***Author response:*** *Data cannot be shared publicly because the data contains sensitive personal data. Public deposition would breach compliance with the protocol approved by the regional research ethics board.*

*According to the Swedish law of ethics in research concerning humans (*[*https://www.riksdagen.se/sv/dokument-lagar/dokument/svenskforfattningssamling/lag-2003460-om-etikprovning-av-forskning-som_sfs-2003-460*](https://www.riksdagen.se/sv/dokument-lagar/dokument/svenskforfattningssamling/lag-2003460-om-etikprovning-av-forskning-som_sfs-2003-460)*) the integrity of the study participants should be protected and data treated accordingly to what is stated in the “informed consent” signed by the participant. In this study, it was stated in the “informed consent” that the participant's personal information would not be shared to third-party recipients or risk to become publicly available.*

*In the case that a researcher or authority would like to access more information, please contact Data Protection Officer (DPO) Sanna Othman, lawyer at the Region of Jämtland -Härjedalen. E-mail address:* [*sanna.othman@regionjh.se*](mailto:sanna.othman@regionjh.se) *. Phone number: +46 63 147586. Postal address: Region Jämtland Härjedalen, Box 654, 831 27 Östersund, Sweden*

*To ensure persistent and long-term data storage and access, digital archiving is provided by the Region of Jämtland-Härjedalen at the hospital of Östersund. Password-protected local servers are used and are accessible only by authorized users.*

Additional Editor Comments:

While the manuscript contains crucial information on iNPH, there is room for enhancement by incorporating the feedback provided by the reviewers.


[Note: HTML markup is below. Please do not edit.]

Reviewers' comments:

Reviewer's Responses to Questions

**Comments to the Author**

1. Is the manuscript technically sound, and do the data support the conclusions?

The manuscript must describe a technically sound piece of scientific research with data that supports the conclusions. Experiments must have been conducted rigorously, with appropriate controls, replication, and sample sizes. The conclusions must be drawn appropriately based on the data presented.

Reviewer #1: Partly

Reviewer #2: Yes

2. Has the statistical analysis been performed appropriately and rigorously?

Reviewer #1: Yes

Reviewer #2: Yes

3. Have the authors made all data underlying the findings in their manuscript fully available?

The [PLOS Data policy](https://emea01.safelinks.protection.outlook.com/?url=http%3A%2F%2Fwww.plosone.org%2Fstatic%2Fpolicies.action%23sharing&data=05%7C02%7C%7C58553a96d171476f6e6808dc17836e35%7C84df9e7fe9f640afb435aaaaaaaaaaaa%7C1%7C0%7C638411100457468343%7CUnknown%7CTWFpbGZsb3d8eyJWIjoiMC4wLjAwMDAiLCJQIjoiV2luMzIiLCJBTiI6Ik1haWwiLCJXVCI6Mn0%3D%7C3000%7C%7C%7C&sdata=mKnGzu0Iyp8QCdJDUrPB8BDrTTHMu2MJaj4M3spLQ7c%3D&reserved=0) requires authors to make all data underlying the findings described in their manuscript fully available without restriction, with rare exception (please refer to the Data Availability Statement in the manuscript PDF file). The data should be provided as part of the manuscript or its supporting information, or deposited to a public repository. For example, in addition to summary statistics, the data points behind means, medians and variance measures should be available. If there are restrictions on publicly sharing data—e.g. participant privacy or use of data from a third party—those must be specified.

Reviewer #1: Yes

Reviewer #2: No

4. Is the manuscript presented in an intelligible fashion and written in standard English?

PLOS ONE does not copyedit accepted manuscripts, so the language in submitted articles must be clear, correct, and unambiguous. Any typographical or grammatical errors should be corrected at revision, so please note any specific errors here.

Reviewer #1: Yes

Reviewer #2: Yes

5. Review Comments to the Author

Please use the space provided to explain your answers to the questions above. You may also include additional comments for the author, including concerns about dual publication, research ethics, or publication ethics. (Please upload your review as an attachment if it exceeds 20,000 characters)

Reviewer #1:

The study is a population-based investigation on the impact of idiopathic normal pressure hydrocephalus (iNPH) on quality of life, depressive symptoms, and functional disability. Through neurological examinations and brain computed tomography scans of 122 participants, the researchers found that those with iNPH reported higher depression scores and lower functioning status compared to those without iNPH, and their health-related quality of life (HRQoL) was poorer. This study is interesting, but I still have some questions as follows:

1. The participants in this study were recruited from a previous cohort study and followed up for two years. The authors should provide detailed inclusion and exclusion criteria for these participants, indicating that they had undergone relevant checks before being included in the study cohort to ensure that they did not have any diseases that could affect the results of this study (for example, whether the participants had excluded the possibility of depressive mood before being included in the study cohort, the authors should elaborate).

***Author response:*** *We have clarified the inclusion and exclusion criteria in the manuscript, page 5, line 104-107. A history of mental illness such as depression was not considered an exclusion criterion.*

2. The authors should detail when they assessed depressive symptoms, functional impairment, and/or HRQoL scores in the participants, ensuring that all scales were evaluated at approximately the same time point to exclude possible errors caused by disease progression.

***Author response:*** *We have clarified when these questionnaires were completed in relation to the clinical examination, see page 7, lines 150-151 and page 8, lines 168-170.*

3. Although Tables 2 and 3 show different data, there is some duplication in the description of lines 196-199 in the results and lines 212-217. The authors should consider integrating them into one paragraph and more accurately describing the related results.

***Author response:*** *We have clarified the headings to table 3 and 4 (previously named Tables 2 and 3).*

4. In Table 4, why did the authors select three different models (A, B, C) for logistic regression? The authors should give reasons; the authors should also provide detailed explanations of the differences in their analysis results to facilitate readers' understanding.

***Author response:***

*Thank you for your advice, we have added more information regarding this in the manuscript, see page 9, line 185- 198; page 14, lines 282-288; and page 15, lines 313-317.*

*For tables 5 and 6 (previously titled table 4 and 5), we utilized multiple logistic regression models to investigate the relationship between several clinically significant parameters and low HRQoL. We started with univariate models and then progressed to more comprehensive multiple models. Model A included all parameters found to be significant in the univariate analyses plus the covariates age and sex.*

*Models B and C were chosen based on clinical reasoning. Recognizing potential interdependencies and overlap among some independent factors in Model A, we introduced two additional models. Model B, alongside age and sex, incorporated imaging signs (Radscale score) and iNPH symptoms (iNPH score)—factors specific to iNPH. From previous analyses (table 3) we saw that the iNPH diagnosis was associated with HRQoL and wanted to explore different aspects of iNPH. Conversely, Model C included depressive symptoms (GDS-15) and functional impairment (mRS), which are more general factors potentially associated with diminished quality of life. We anticipated that this approach would offer valuable insights by distinguishing between analyses based on distinct sets of predictors.*

Reviewer #2:

This study conducted an association analysis of anxiety symptoms, functional status and quality of life among iNPH patients in a population-based sample. In general, the sample size and analysis angle included in this article were acceptable. However, the following aspects need to be further improved by the author:

1. Please clarify the grouping criteria in detail. How to clearly define "iNPH patients" and "unlikely iNPH patients".

***Author response:*** *Thank you. We have clarified this in the revised manuscript and included a new table (Table 1). Please see page 7, lines 133-140.*

2. All participants underwent head imaging, and I recommend supplementating with representative imaging images.

***Author response:*** *Thank you for your suggestion, we have included two figures as supporting information S1 Fig., S2 Fig., see page 26, lines 565-568.*

3. The study sample is the population of patients enrolled in 2014 and followed up in 2017. Whether the authors can further follow-up up to 2024 makes the conclusion more convincing.There may be some new discoveries.

***Author response:*** *Thank you for your suggestion. We agree, it would be interesting to have a longer follow-up, but we did not plan for this and have not applied to the ethical board. We may take up on your advice in the future.*

6. PLOS authors have the option to publish the peer review history of their article ([what does this mean?](https://emea01.safelinks.protection.outlook.com/?url=https%3A%2F%2Fjournals.plos.org%2Fplosone%2Fs%2Feditorial-and-peer-review-process%23loc-peer-review-history&data=05%7C02%7C%7C58553a96d171476f6e6808dc17836e35%7C84df9e7fe9f640afb435aaaaaaaaaaaa%7C1%7C0%7C638411100457468343%7CUnknown%7CTWFpbGZsb3d8eyJWIjoiMC4wLjAwMDAiLCJQIjoiV2luMzIiLCJBTiI6Ik1haWwiLCJXVCI6Mn0%3D%7C3000%7C%7C%7C&sdata=wLVq3XiWzmaP3FLoZ%2BaE%2B1GPbFLoAjHIqN18VIS7YJE%3D&reserved=0)). If published, this will include your full peer review and any attached files.

If you choose “no”, your identity will remain anonymous but your review may still be made public.

**Do you want your identity to be public for this peer review?** For information about this choice, including consent withdrawal, please see our [Privacy Policy](https://emea01.safelinks.protection.outlook.com/?url=https%3A%2F%2Fwww.plos.org%2Fprivacy-policy&data=05%7C02%7C%7C58553a96d171476f6e6808dc17836e35%7C84df9e7fe9f640afb435aaaaaaaaaaaa%7C1%7C0%7C638411100457468343%7CUnknown%7CTWFpbGZsb3d8eyJWIjoiMC4wLjAwMDAiLCJQIjoiV2luMzIiLCJBTiI6Ik1haWwiLCJXVCI6Mn0%3D%7C3000%7C%7C%7C&sdata=knx62NBPq26aOCqqvxEpX8gwA5jGVnw6dEtWUJT5nNo%3D&reserved=0).

Reviewer #1: No

Reviewer #2: No
